# Prognostic value of lncRNAs in lung carcinoma: a meta-analysis

**DOI:** 10.18632/oncotarget.21096

**Published:** 2017-09-20

**Authors:** Fan Fan, Zhengqiu Zhu, Chao Gao, Yun Liu, Baoqing Wang, Ziquan Wang, Jifeng Feng

**Affiliations:** ^1^ Department of Chemotherapy, Nanjing Medical University Affiliated Cancer Hospital, Jiangsu Cancer Hospital, Jiangsu Institute of Cancer Research, Nanjing 210009, China; ^2^ Department of Chemotherapy, The No.2 Affiliated Hospital of Xuzhou Medical University, Xuzhou 221000, China; ^3^ Department of Chemotherapy, The Affiliated Hospital of Xuzhou Medical University, Xuzhou 221000, China

**Keywords:** lncRNA, lung cancer, prognosis

## Abstract

Many different long non-coding RNAs (lncRNAs) have been reported to be abnormally expressed in lung carcinoma and may thus serve as prognostic biomarkers for this disease. We conducted this meta-analysis, which included a total of 30 studies identified via searches of PubMed, Embase, Medline, and Web of Science and included 2912 patients from China (28), Germany (1), and Japan (1), to investigate the prognostic value of different lncRNAs in lung carcinoma. The results revealed that lncRNA transcription levels were significantly associated with overall survival in lung cancer patients (HR:1.46, 95% CI: 1.16–1.83, *P* = 0.000). However, lncRNA transcription levels were not associated with progression-free survival (PFS) (HR: 1.55, 95% CI: 0.50–4.80, *P* = 0.449). Further analysis showed that high lncRNA transcription levels were significantly associated with tumour-node-metastasis (TNM) stage (III/IV vs I/II: RR = 1.339, 95% CI: 1.046–1.716, *P* = 0.012), lymph node metastasis (positive vs negative: RR = 1.442, 95% CI: 1.103–1.885, *P* = 0.007), and distant metastasis (yes vs no: RR = 3.187,95% CI: 1.393–7.294, *P* = 0.006). Taken together, the results of our present meta-analysis revealed that lncRNAs may be useful prognostic markers for lung carcinoma and may also have value as biomarkers for TNM stage, lymph node metastasis and distant metastasis.

## INTRODUCTION

Lung cancer is the leading cause of cancer-related death among males in both developed and developing countries and has surpassed breast cancer as the leading cause of cancer-related death among females in developed countries [1]. Lung cancers are generally classified as non-small cell lung cancer (NSCLC) or small cell lung cancer (SCLC), which are the two main types of lung cancer. NSCLC, which comprises adenocarcinoma, squamous cell carcinoma and large cell carcinoma, is the most prominent type of lung cancer and accounts for approximately 85% of all lung cancer cases [2]. SCLC is an aggressive type of lung cancer with neuroendocrine features that grows more rapidly and recurs more frequently than NSCLC [3]. Surgery, chemotherapy, and radiation therapy have improved the prognosis of lung cancer; however, despite recent advances in lung cancer diagnostic strategies and treatments, particularly, advances in EGFR and ALK gene detection strategies and targeted therapies, the overall survival (OS) of lung cancer patients remains poor. Therefore, studies aiming to identify more sensitive and specific biomarkers for the prognosis of these patients are desired and urgently needed.

Evidence gathered in recent decades indicates that at least 90% of the total mammalian genome is actively transcribed [4]. However, only approximately 1.5% of the genome contains protein-coding genes [5]. Non-protein-coding RNA (ncRNA) transcripts constitute > 98% of the mammalian transcriptome and were once considered “transcription noise”. LncRNAs are a class of non-coding RNAs greater than 200 nucleotides in length [6] that have been shown to regulate many key biological functions [7], such as cell differentiation, fate determination, cell proliferation, and cell migration [8, 9].

Aberrant lncRNA expression has been noted in many types of cancer. For example, lncRNA HOTAIR levels are elevated in many types of cancer, including primary and metastatic breast cancer [10], colorectal carcinoma [11] and gastrointestinal stromal cancer [12], and in most cases, high HOTAIR expression is associated with poor patient survival. High levels of the lncRNA HULC have been observed in hepatocellular carcinoma (HCC) tissues, as well as in metastatic tumours derived from the liver but not those from the lymph nodes, indicating that this lncRNA is specific to malignant cells located in the liver [13]. Further, over-expression of the lncRNA BRAF-activated non-coding RNA (BANCR) has been observed in non-small cell lung cancer cells and has been demonstrated to be significantly associated with metastasis [14].

Correlation analyses have shown that lncRNAs have the potential to serve as diagnostic or prognostic markers in lung cancer patients. HMlincRNA717 may be a prognostic biomarker for NSCLC, as its down-regulation is suggestive of poor prognosis in patients with this disease [15]. Moreover, high lncRNA ZXF2 levels are associated with poor OS and thus, it may be an important prognostic biomarker in patients with lung adenocarcinoma metastasis [16]. Additionally, high MVIH expression has been reported to be associated with a relatively poor prognosis in NSCLC patients [17].

To date, multiple lncRNAs, including MALAT1, UCA1, SPRY4-IT1, CCAT2, AFAP1-AS1 and BANCR, have been confirmed to be promising prognostic indicators in lung cancer patients. However, because of between-study differences in sample size and research methodology, the results may differ among the studies. In addition, such results may be insufficient with respect to the conclusions regarding the value of lncRNAs in patients with lung cancer. To gain further insights into the prognostic value of lncRNAs in these patients, we conducted a meta-analysis to determine the prognostic value of abnormally expressed lncRNAs.

## RESULTS

### Characteristics of the eligible studies

**Figure 1:**
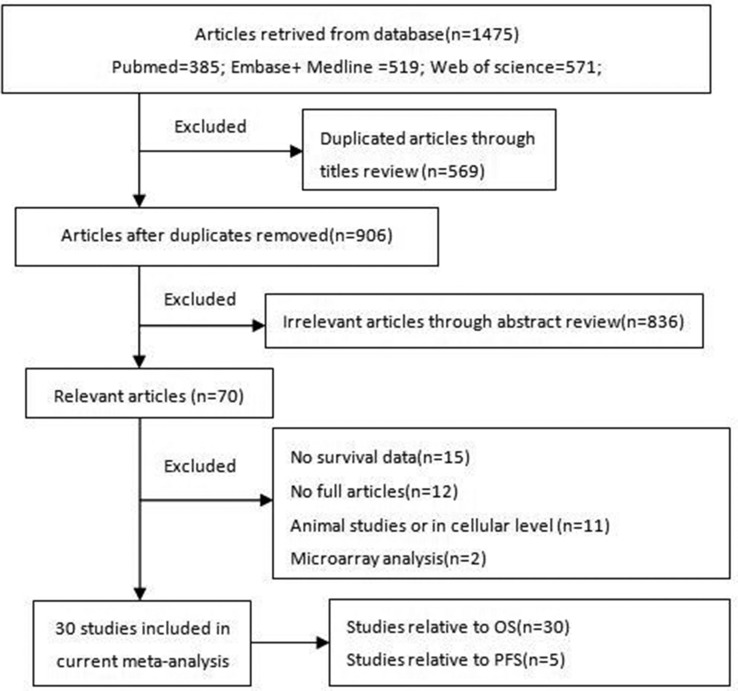
Flowchart of the literature search and selection steps Abbreviations: lncRNAs, long noncoding RNAs; OS, overall survival; PFS, progress-free survival.

We retrieved 1475 articles from PubMed, Embase, Medline, and Web of Science, as shown in the corresponding flow diagram. After reviewing the titles of the manuscripts, we excluded 569 duplicate articles, as well as 836 articles reporting irrelevant or insufficient data. We subsequently assessed a total of 70 relevant articles and excluded 40 studies, including 15 studies lacking survival data, 12 lacking full articles, 11 involving animals or cellular models, and 2 in which only microarray analyses were performed, according to our exclusion criteria. The detailed process by which the studies were screened is shown in Figure 1.

According to the inclusion and exclusion criteria, 30 studies including 2912 patients from China (28), Germany (1), and Japan (1) were included in the current meta-analysis [14–43]. The characteristics of the 30 studies included in the present meta-analysis are summarised in Table 1. OS and progression-free survival (PFS) were estimated as survival outcome measures in 100% (30/30) and 16.7% (5/30) of the above studies, respectively.

**Table 1:** Characteristics of the studies included in the meta-analysis

Studies	Region	LncRNAs	Tumour type	Expression	Method	Study population (high/low)	Cut-off	Follow-up (month)	Outcome	Quality score (%)
Zhang et al. [18] 2016	China	H19	NSCLC	up-regulation	qPCR	35/35	median	60	OS	83.3%
Sun et al. [14] 2014	China	BANCR	NSCLC	down-regulation	qPCR	53/60	fold change	40	OS/PFS	83.3%
Zhang et al. [19] 2014	China	ZXF1	NSCLC	up-regulation	qRT-PCR	43/19	ratio	36	OS	73.8%
Sun et al. [20] 2014	China	SPRY4-IT1	NSCLC	down-regulation	qRT-PCR	60/61	fold change	40	OS/PFS	73.8%
Yang et al. [21] 2014	China	PVT1	NSCLC	up-regulation	qRT-PCR	65/17	median	60	OS	83.3%
Wan et al. [22] 2016	China	PCAT6	NSCLC	up-regulation	qRT-PCR	28/26	mean	60	OS	73.8%
Zhou et al. [23] 2016	China	AB209630	NSCLC	down-regulation	qRT-PCR	103/35	fold change	60	OS	73.8%
Xie et al. [15] 2014	China	HMlincRNA717	NSCLC	down-regulation	qRT-PCR	49/69	mean	80	OS	78.6%
Nie et al. [24] 2016	China	UCA1	NSCLC	up-regulation	RT-PCR	39/73	Youden index	80	OS	83.3%
Luo et al. [25] 2014	China	CARLo-5	NSCLC	up-regulation	qRT-PCR	29/33	median	80	OS	73.8%
Zang et al. [26] 2016	China	LINC01133	NSCLC	up-regulation	qRT-PCR	34/34	fold change	40	OS/PFS	73.8%
Nie et al. [17] 2014	China	MVIH	NSCLC	up-regulation	qPCR	21/21	median	40	OS	73.8%
Sun et al. [27] 2016	China	NEAT1	NSCLC	up-regulation	qRT-PCR	67/29	fold change	40	OS	73.8%
Wang et al. [28] 2016	China	PVT-1	NSCLC	up-regulation	qRT-PCR	74/71	mean	60	OS	78.6%
Zhao et al. [29] 2016	China	SBF2-AS1	NSCLC	up-regulation	qRT-PCR	80/94	median	70	OS	83.3%
Zhang et al. [30] 2014	China	TUG1	NSCLC	down-regulation	qRT-PCR	96/96	median	60	OS	73.8%
Wang et al. [31] 2015	China	LINC01207	NSCLC	up-regulation	qRT-PCR	49/11	fold change	60	OS	78.6%
Deng et al. [32] 2015	China	AFAP1-AS1	NSCLC	up-regulation	qRT-PCR	66/55	NA	60	OS	76.2%
Wang et al. [33] 2015	China	UCA1	NSCLC	up-regulation	qRT-PCR	36/24	median	80	OS	83.3%
Zhang et al. [34] 2015	China	LINC01133	LSCC	up-regulation	qRT-PCR	NA	median	60	OS	73.8%
Yang et al. [16] 2015	China	ZXF2	NSCLC	up-regulation	qRT-PCR	27/13	fold change	36	OS	73.8%
Hou et al. [35] 2014	China	Sox2ot	NSCLC	up-regulation	qRT-PCR	NA	fold change	60	OS	83.3%
Han et al. [36] 2013	China	GAS6-AS1	NSCLC	down-regulation	qRT-PCR	25/25	mean	60	OS	83.3%
Han et al. [37] 2015	China	PANDAR	NSCLC	down-regulation	qRT-PCR	70/70	mean	60	OS	83.3%
Li et al. [38] 2016	China	AGAP2-AS1	NSCLC	up-regulation	qPCR	40/40	mean	40	OS/PFS	73.8%
Schmidt et al. [39] 2011	Germany	MALAT1	NSCLC	up-regulation	ISH	NA	NA	56	OS	72.7%
Hiroshi et al. [40] 2014	Japan	HOTAIR	SCLC	up-regulation	qRT-PCR	8/10	median	125	OS	71.4%
Huang et al. [41] 2016	China	PVT1	SCLC	up-regulation	qRT-PCR	60/60	median	96	OS	83.3%
Chen et al. [42] 2016	China	CCAT2	SCLC	up-regulation	qRT-PCR	56/56	median	60	OS	83.3%
Liu et al. [43] 2016	China	AK09398	SCLC	up-regulation	qRT-PCR	66/52	median	80	OS/PFS	73.8%

### Prognosis

**Figure 2:**
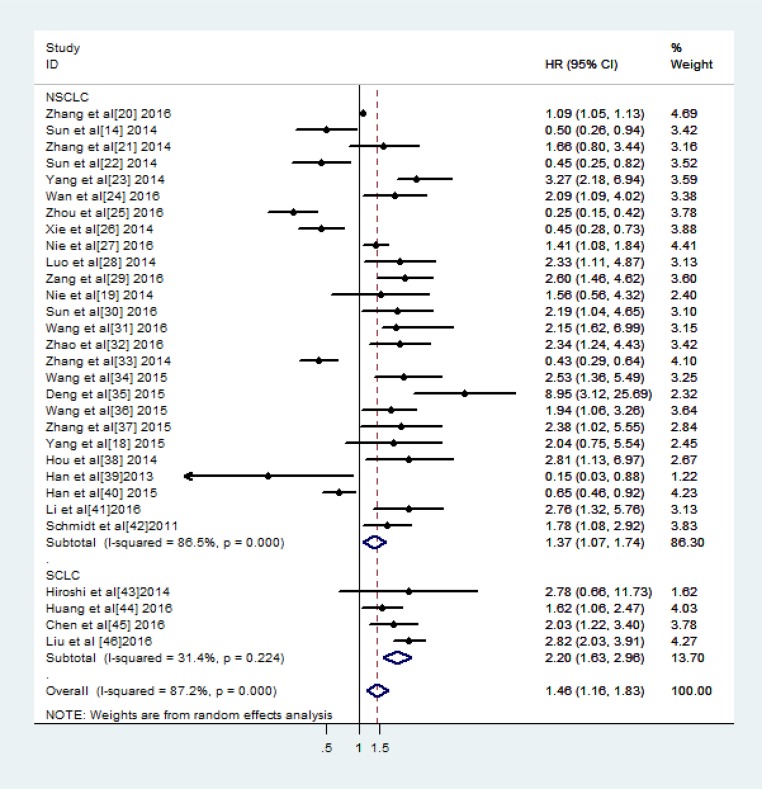
Meta-analysis of the pooled HRs for OS in different pathological types of lung cancer with high lncRNAs expression

A total of 30 studies assessed the relationships between the expression of 26 different lncRNAs and OS in 2912 patients with lung cancer. Data pertaining to the hazard ratios (HRs) and corresponding 95% confidence intervals (CIs) for OS were extracted from the included studies, and HRs > 1 were suggestive of a poor prognosis [44]. The estimated pooled HR for all the studies showed that lncRNA transcription levels were significantly associated with OS in lung cancer patients (HR: 1.46, 95% CI: 1.16–1.83, *P =* 0.000, random-effects model) (Figure 2); however, significant between-study heterogeneity was noted with respect to the relationship between lncRNA expression and OS (I^2^ = 87.2%, *P =* 0.000).

Twenty-six articles reported data pertaining to the relationship between lncRNAs and OS in NSCLC patients, and four articles reported data pertaining to the above relationship in SCLC patients. Decreased BANCR, SPRY4-IT1, AB209630, HMlincRNA717, SBF2-AS1, TUG1, ZXF2, GAS6-AS1 and PANDAR expression and increased H19, ZXF1, PVT1, PCAT6, UCA1, CARLo-5, LINC01133, MVIH, NEAT1, LINC01207, AFAP1-AS1, Sox2ot, MALAT1, AGAP2-AS1, HOTAIR, AK09398 and CCAT2 expression were associated with a poor prognosis. We subsequently performed a subgroup analysis, in which we assessed the relationship between lncRNA expression and OS in patients with specific types of tumours. The results of the analysis showed that lncRNA expression was significantly associated with OS in patients with NSCLC (HR:1.37, 95% CI: 1.07–1.74, *P =* 0.012) and in those with SCLC (HR:2.20, 95% CI: 1.63–2.97, *P =* 0.000). Thus, we speculated that the ability of lncRNA transcription levels to predict OS is affected by the tumour type.

**Figure 3:**
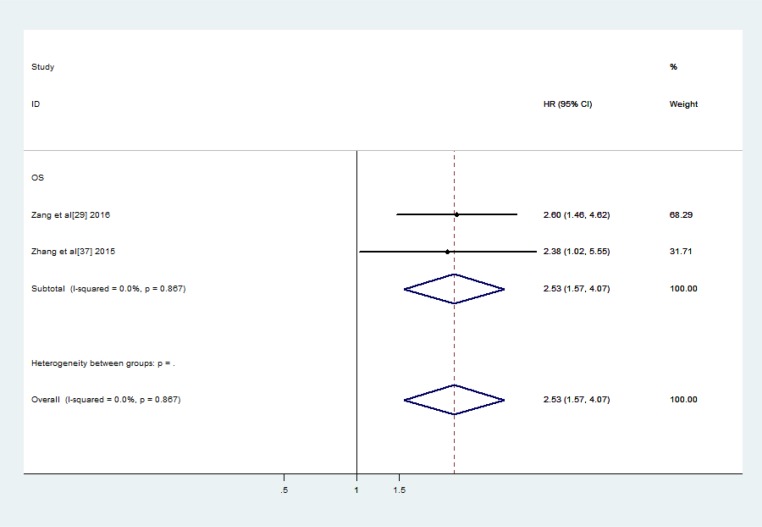
Forest plots of the studies evaluate the HRs for up-regulated LINC01133 expression and the OS of lung carcinoma patients

**Figure 4:**
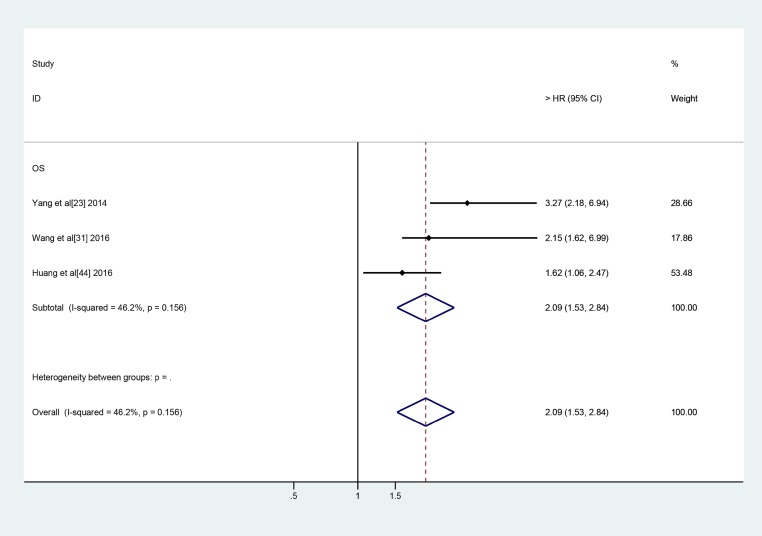
Forest plots of the included studies evaluate the HRs of PVT1 and OS

**Figure 5:**
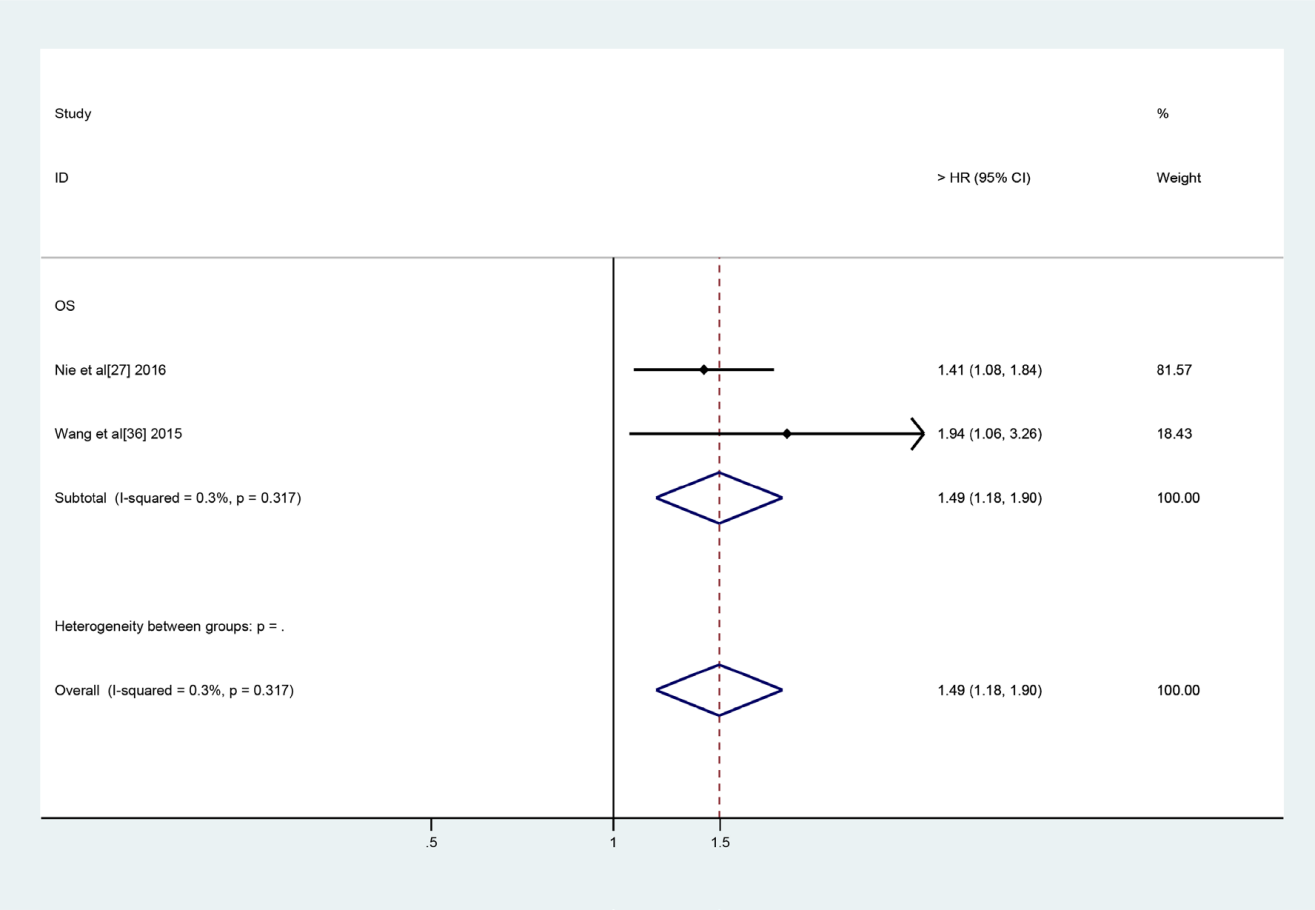
Forest plots of studies evaluate the HRs for UCA1 expression and the OS of lung carcinoma patients

All 26 of the above-mentioned lncRNAs, with the exception of LINC01133, PVT-1 and UCA1, were investigated in one study. LINC01133 and UCA1 were investigated in two studies, while PVT-1 was investigated in three studies. Thus, we subsequently conducted a meta-analysis to assess the relationships between LINC01133, UCA1 and PVT-1 expression and OS in lung cancer patients. We noted no significant between-study heterogeneity with respect to the above relationships. Therefore, we applied the fixed effects model, which showed that high LINC01133 expression was associated with poor OS (HR: 2.53, 95% CI: 1.57–4.07, *P =* 0.000) (Figure 3). Additionally, we found that PVT-1 and UCA1 were predictors of a short OS (PVT-1: HR: 2.09, 95% CI: 1.53–2.84, *P =* 0.000) (UCA1: HR: 1.49, 95% CI: 1.18–1.90, *P =* 0.001) (Figures 4 and 5).

**Figure 6:**
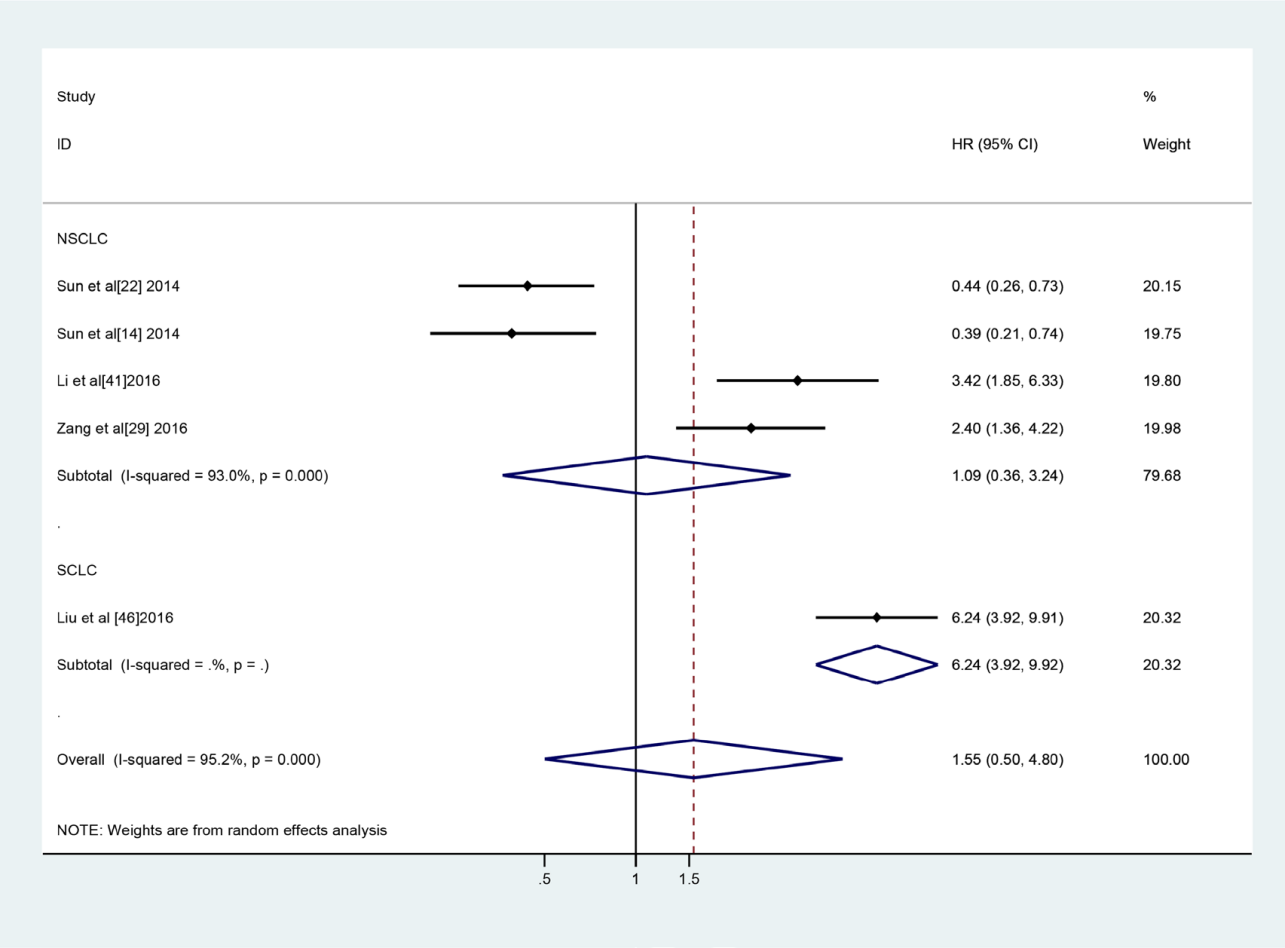
Forest plot of the studies evaluates the HRs for the expression of lncRNAs and PFS in lung cancer

A total of five studies involving 500 patients investigated the relationships between 5 different lncRNAs and PFS. High AK09398, AGAP2-AS1 and LINC01133 expression was associated with a poor prognosis, and SPRY4-IT1 and BANCR down-regulation was associated with a relatively poor prognosis. However, further analysis showed that lncRNA expression levels were not associated with PFS in lung cancer patients (HR: 1.55, 95% CI: 0.50–4.80, *P =* 0.449) (Figure 6). Sensitivity analysis and assessments of publication bias specific to the relationship between lncRNA expression and PFS were not performed because only a small number of articles regarding the relationship were included in the meta-analysis.

### Correlations between lncRNA expression and lung cancer clinicopathological characteristics

The association between lncRNA expression and clinicopathological characteristics was analysed, and corresponding relative risk (RR) values were determined. A RR > 1 implied that lncRNA expression was associated with a particular parameter. High lncRNA transcription levels were significantly associated with TNM stage (III/IV vs I/II: RR = 1.339, 95% CI: 1.046–1.716, *P =* 0.012), lymph node metastasis (positive vs negative: RR = 1.442, 95% CI: 1.103–1.885, *P =* 0.007), and distant metastasis (yes vs no: RR = 3.187, 95% CI: 1.393–7.294, *P =* 0.006) in lung carcinoma. However, we noted no significant correlations between lncRNA expression and tumour histological classification (SCC vs AD: RR = 1.013, 95% CI: 0.891–1.153, *P =* 0.839), tumour histological grade (poor vs well: RR = 0.951, 95% CI: 0.689–1.312, *P =* 0.759), tumour size (> 3 vs ≤ 3, RR = 1181, 95% CI: 0.883–1.580; > 5 vs ≤ 5: RR = 1.313, 95% CI: 0.904–1.905), or smoking status (yes vs no, RR = 1.040, 95% CI: 0.918–1.179). Additionally, we noted no associations between lncRNA expression and other characteristics, such as age and sex (Table 2).

**Table 2:** Associations between lncRNA levels and lung cancer clinicopathologic characteristics

Characteristic	Number of studies	Pooled RR (95% CI)	*P*	Heterogeneity test		References
				*I*^*2*^	*P*	
Age (> 50 vs ≤ 50)	3	1.086 (0.907,1.301)	0.370	0.0%	0.823	32, 41, 42
(> 55 vs ≤ 55)	3	1.057 (0.874,1.277)	0.570	0.0%	0.985	28, 29, 43
(> 60 vs ≤ 60)	7	1.013 (0.868,1.182)	0.871	0.0%	0.654	16, 19, 21, 22, 24, 33, 36
(> 65 vs ≤ 65)	6	0.992 (0.822,1.198)	0.937	0.0%	0.825	14, 17, 20, 26, 27, 38
Gender (male vs female)	19	0.983 (0.906,1.066)	0.682	0.0%	0.834	14, 16, 17, 19, 20, 21, 22, 24, 26, 27, 28, 29, 32, 33, 36, 38, 41, 42, 43
Histological grade (poor vs well)	8	0.951 (0.689,1.312)	0.759	70.1%	0.001	16, 18, 19, 22, 27, 28, 30, 36
Histological classification (SCC vs AD)	11	1.013 (0.891,1.153)	0.839	35.0%	0.119	14, 18, 20, 15, 26, 17, 27, 29, 30, 36, 38,
Tumour size (> 3 vs ≤ 3)	11	1.181 (0.883,1.580)	0.262	74.9%	0.000	15, 16, 18, 21, 22, 24, 27, 28, 29, 30, 33, 36
(> 5 vs ≤ 5)	6	1.313 (0.904,1.905)	0.152	82.0%	0.000	14, 17, 20, 26, 38, 41
TNM Stage (III/IV vs I/II)	13	1.339 (1.046,1.716)	0.012	86.5%	0.000	14, 15, 16, 17, 18, 19, 20, 21, 22, 24, 26, 27, 28, 30, 32, 33, 38
Lymph node metastasis (positive vs negative)	20	1.442 (1.103,1.885)	0.007	86.7%	0.000	14, 15, 16, 17, 18, 19, 20, 21, 22, 26, 27, 28, 29, 30, 32, 33, 38, 41, 42, 43
Distant metastasis (yes vs no)	4	3.187 (1.393,7.294)	0.006	83.8%	0.000	32, 41, 42, 43
Smoking status (yes vs no)	18	1.040 (0.918,1.179)	0.536	58.6%	0.001	14, 16, 17, 18, 19, 20, 22, 24, 26, 27, 28, 30, 32, 36, 38, 42, 43

### Publication bias and sensitivity analysis

**Figure 7:**
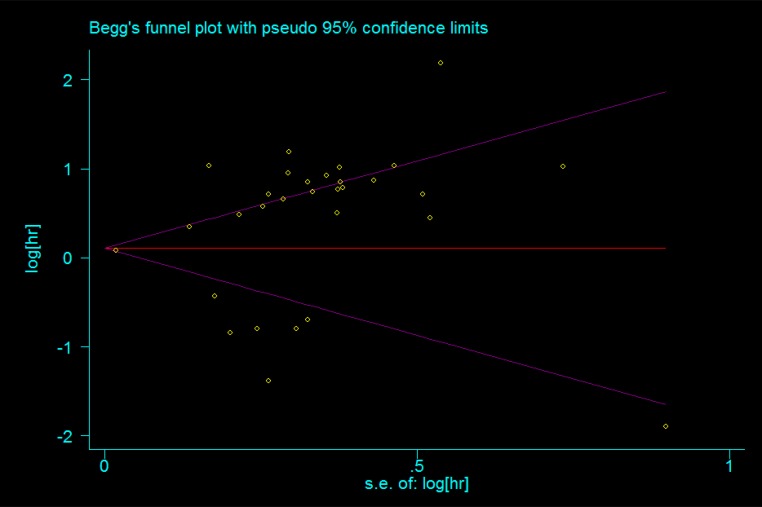
Publication bias of the association between the expression of lncRNAs and the OS of patients with lung carcinoma was assessed by Begg’s funnel plots

**Figure 8:**
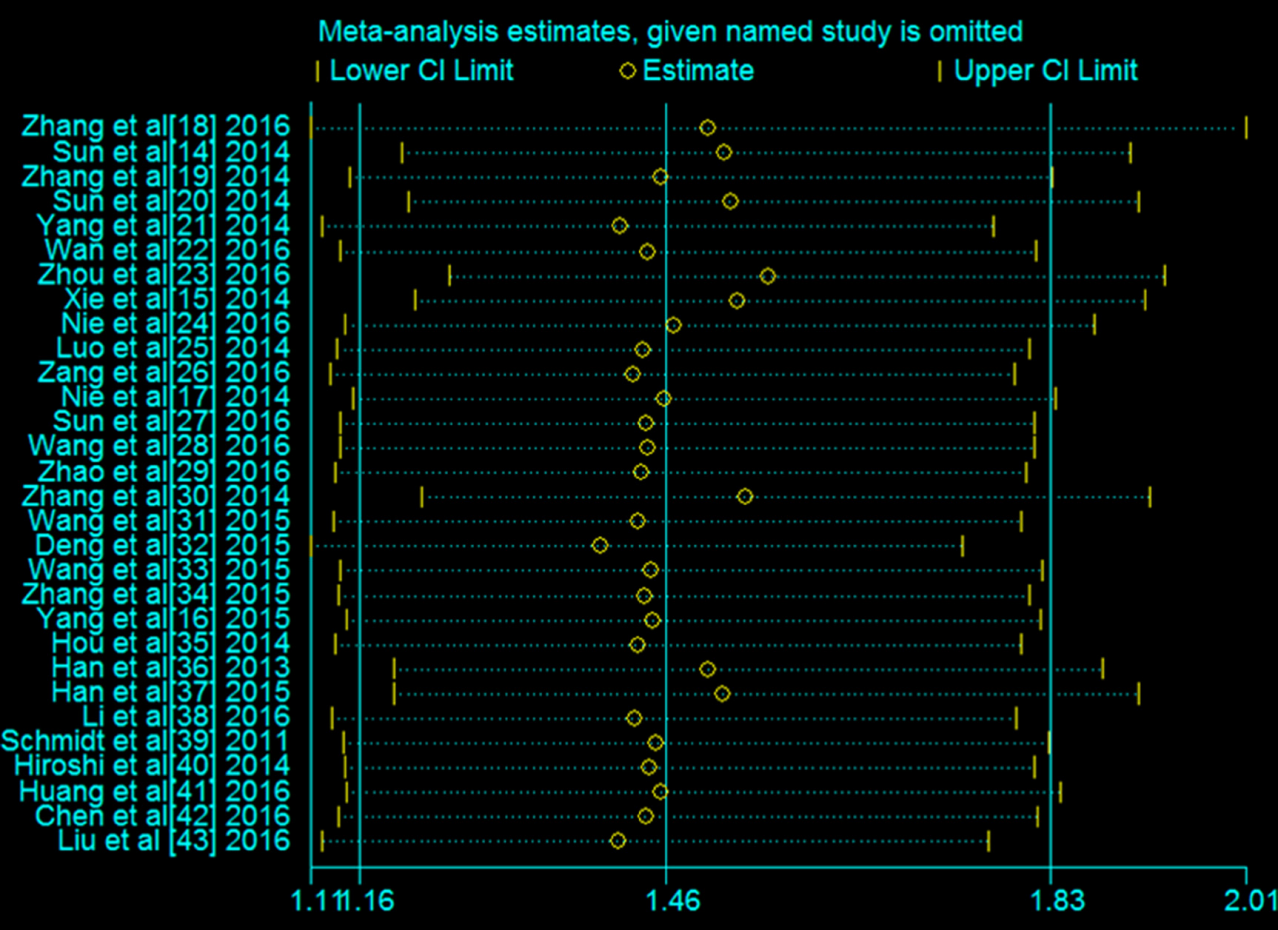
Sensitivity analysis of the association between the expression of lncRNAs and the OS of patients with lung carcinoma

Publication bias was evaluated using Begg’s funnel plots, which showed that no significant publication bias was present in the studies included in the analysis (Figure 7).Sensitivity analysis indicated that no single study affected the overall results of the analysis (Figure 8) and thus confirmed the stability of the results.

## DISCUSSION

In 2000, Weinberg and Hanahan proposed that the hallmarks of cancer comprise six biological capabilities acquired during the multistep development of human tumours [45]. Underlying these hallmarks is genomic instability, which generates the genetic diversity that expedites the acquisition of these capabilities [46]. Over the past several decades, tumour genome sequencing has enabled the documentation of thousands of DNA mutations and other genomic alterations. Edwin Wang et al. [47] applied mathematical modelling tools to represent cancer hallmarks and model genome sequencing data to predict cancer clonal evolution and associated clinical phenotypes, called cancer hallmark networks. Among these networks, the mechanisms of cancer aetiology attributed to the signalling pathways of some cancer hallmarks are closely intertwined. Furthermore, Gao [48] developed robust combinatory cancer hallmark-based gene signature sets (CSS) and demonstrated that these sets significantly improved the predictive accuracy of prognosis in patients with stage II Colorectal Cancer. Thus, with holistic clarity of networks, it will be possible to predict cancer prognosis and precisely understand how and why treatment regimens and specific antitumour drugs succeed or fail.

Traditionally, most studies investigating carcinoma mechanisms have focused on protein-coding genes. Surprisingly, the ENCODE project has revealed that 87.3% of the human genome is actively transcribed, although only < 3% encodes proteins [49]. Scientists are able to investigate gene expression in transcribed but not translated genes [50], such as noncoding RNAs (ncRNAs) without protein-coding ability [51]. A newly discovered class of non-coding genes known as lncRNAs have been shown to be involved in regulating gene expression, chromatin remodelling, transcription, post-transcriptional RNA processing and cancer progression [52].

Accumulating evidence has shown that lncRNAs play important roles in the development and progression of multiple cancers [53]. For example, maternally expressed gene 3 (MEG3) expression levels are markedly reduced in HCC tissues and cell lines, and loss of MEG3 gene expression is associated with promoter region hypermethylation in HCC [54]. Importantly, enforced MEG3 expression in HCC cells significantly induces cell apoptosis. Additionally, the lncRNA colon cancer-associated transcript 2 (CCAT2) enhances WNT activity by binding to TCF7L2, a pivotal transcription factor in the WNT signalling pathway, and facilitates MYC activity, thereby enhancing cancer cell invasion and metastasis [55]. Li et al. [56] found that ANCR modulates EZH2 stability and thus plays a role in breast cancer cell invasion and metastasis. Specifically, they found that ANCR facilitates breast cancer progression and metastasis mainly by decreasing EZH2 stability.

Functional studies have revealed a broad spectrum of mechanisms used by lncRNAs to execute their functions, and they have shown that lncRNAs have some associations with cancer hallmarks. Thus, lncRNAs could offer a number of advantages as diagnostic and prognostic markers and also as novel specific therapeutic targets, as supported by increasing evidence. For example, uc.73a expression is lower in CRC tissues than in corresponding noncancerous tissues [57]. Patients with low uc.73a expression have relatively poor OS compared with those with high uc.73a expression. GAS5, an lncRNA of approximately 650 bp, has been shown to be significantly down-regulated in gastric cancer tissues compared with corresponding normal tissues [58], and this decreased expression has been shown to be associated with a large tumour size, advanced pathologic stage, and poorer DFS and OS. Additionally, Chen et al. [59] have shown that patients with cervical squamous cell cancer with high lncRNA CCAT2 expression have poorer OS and PFS than those with lower lncRNA CCAT2 expression.

Thus, we conducted this meta-analysis, which was the first to investigate the association between lncRNA expression and prognosis in lung carcinoma, to identify biomarkers for lung carcinoma.

A total of 26 different lncRNAs were assessed in the 30 articles included in the present meta-analysis. The expression of BRANCR, SPRY4-IT1, AB209630, HMlinc717, TUG1, GAS6-AS1 and PANDAR was down-regulated, while the expression of other lncRNAs was up-regulated in lung cancer patients. Our meta-analysis showed that high lncRNA transcription levels represented a significant risk factor for OS, after pooling of the HRs and *P*-values. However, we noted no significant association between lncRNA transcription levels and PFS.

Most of the lncRNAs were evaluated in a single study. LINC01133 and UCA1 were assessed in two studies, while PVT-1 was investigated in three studies. The pooled HRs for the relationships between these three lncRNAs and OS were 2.53 (95% CI: 1.57–4.07, I^2^ = 0.0%, *P =* 0.867), 1.49 (95% CI: 0.64–2.69, I^2^ = 0.3%, *P =* 0.317) and 2.09 (95% CI: 1.53–2.84, I^2^ = 46.2%, *P =* 0.156), respectively.

LINC01133, which was found to be differentially expressed between LSCC and LAD, according to the results of a data mining analysis using the GEO database and an Affymetrix HG-U133 Plus 2.0 microarray, was up-regulated in LSCC but not in LAD. Additionally, Zhang et al. [34] have observed decreased survival in patients with high LINC01133 expression compared with those with low LINC01133 expression levels, suggesting that LINC01133 may be an effective biomarker for LSCC. These authors have also shown that LINC01133 is over-expressed in NSCLC and that it is correlated with poor prognosis in patients with this disease. Additionally, their study has provided the first evidence that LINC01133 exerts oncogenic effects in human NSCLC cells by interacting with EZH2 and LSD1 and repressing KLF2, P21 and E-cadherin expression.

PVT1 was originally identified as a common retroviral integration site in murine leukaemia virus (MLV)-induced T lymphoma [60]. Accumulating evidence suggests that PVT1 is over-expressed in many types of human cancers, including ovarian cancer, breast cancer, HCC, bladder cancer and gastric cancer [61]. Yang [21] et al. have found that the expression of lncRNA PVT1 is up-regulated in NSCLC and that it is positively correlated with histological grade and lymph node metastasis, and similar findings have also been noted in a study conducted by Wang et al. [29]. Huang et al. [44] have confirmed that PVT1 is over-expressed in SCLC tissues and cell lines. All three of these studies have shown that PVT1 expression is an independent prognostic indicator with respect to OS in NSCLC and SCLC patients.

UCA1, which is also known as urothelial carcinoma associated 1, is a lncRNA that was originally identified in bladder transitional cell carcinoma [62]. Wang et al. [33] and Nie et al. [24] have shown that UCA1 over-expression is associated with poor survival and that it may be an independent prognostic factor for OS in NSCLC patients.

Moreover, we evaluated the correlation between lncRNA transcription levels and the main lung carcinoma clinicopathological parameters. We found that high lncRNA transcription levels were significantly associated with a high TNM stage, lymph node metastasis, and distant metastasis. However, we noted no significant correlation between lncRNA transcription levels and histological classification, histological grade, tumour size, smoking status, age or sex.

This meta-analysis had several limitations. First, as papers with negative results are published less frequently than those with positive results, our results may have been affected by publication bias. Second, we calculated HRs ourselves based on data provided in the papers, which may not have provided the most accurate estimate of the HR possible, as most of the time these data were extracted from Kaplan-Meier curves. However, this practice has not been shown to yield results significantly different from direct methods of HR estimation [63]. Third, the criteria used to determine whether specific lncRNAs were expressed at high levels differed among the studies included in the analysis. Fourth, it was interesting to find that the majority of the eligible studies were conducted in Asia, especially in China. The data collection may be incomplete because data from non-English language papers were not included. Thus, we need more clinical studies including individuals of different races to prove our findings.

In conclusion, our analysis showed that lncRNAs may be used as biomarkers for lymph node metastasis and distant metastasis. Furthermore, lncRNAs may represent prognostic biomarkers for lung carcinoma. However, additional comprehensive, large-scale, and high-quality studies should be conducted to verify our findings and confirm the clinical utility of lncRNAs as prognostic markers in lung carcinoma.

## MATERIALS AND METHODS

### Search strategy

Two authors independently searched PubMed, Embase, Medline, and Web of Science to retrieve all relevant articles regarding the prognostic value of lncRNA in lung cancer. The published data were searched in accordance with the systematic review and meta-analysis guidelines of tumour marker prognostic studies (REMARK), the Preferred Reporting Items for Systematic Reviews and Meta-Analysis (PRISMA) Statement issued in 2009 as well as the checklist of the Dutch Cochrane Centre represented by MOOSE [64–66]. Both MeSH terms and free-text words were utilised to increase the sensitivity of the search, which was performed using the following specific terms: (“Long noncoding RNA”, “lncRNA”, “LincRNA”, “Long ncRNA”, “Long intergenic non-coding RNA”) AND (“Pulmonary Neoplasms”, “Lung Neoplasm”, “Lung Cancer”, “Neoplasms, Lung”, “Neoplasm, Lung”, “ Neoplasms, Pulmonary”, “Neoplasm, Pulmonary”, “Pulmonary Neoplasm”, “Cancer, Lung”, “Cancers, Lung”, “Lung Cancers”, “Pulmonary Cancer”, “Cancer, Pulmonary”, “Cancers, Pulmonary”, “Pulmonary Cancers”, “Cancer of the Lung”, “Cancer of Lung”). The literature covered was restricted to publications in English. Their reference lists were searched manually to identify additional relevant studies.

### Eligibility criteria

All the included studies were systematically reviewed and evaluated based on the reporting checklists MOOSE, REMARK and PRISMA [64–66]. The following studies were eligible for inclusion in the analysis: studies involving patients with a pathological diagnosis of lung cancer, regardless of TNM stage; studies in which lncRNA expression levels in tumour and adjacent non-tumour tissues from lung cancer patients were determined using quantitative reverse transcription polymerase chain reaction or microarray analysis; studies in which the prognostic value of one lncRNA was investigated; studies in which the relationship between lncRNA expression and survival was examined; and studies providing sufficient data for the estimation of HRs and the corresponding 95% CIs for survival rates. Time-to-event data, which were used to determine survival rates (Kaplan-Meier curves), were extracted to calculate these HRs using previously described methods. All eligible studies were carefully assessed by the same two authors, and disagreements were resolved through discussion with a third reviewer (Baoqing Wang). Inter-reviewer agreement was assessed using Cohen’s kappa coefficient. Disagreement was resolved by consensus.

### Quality assessment

To determine the quality of a paper, all eligible studies were scored as previously reported [67]. The assessment was performed by two authors who reached an agreement on all items assessed. The categories of score assessment included the scientific design (five items: study objective definition, study design, outcome definition, statistical consideration, statistical method and test description), laboratory methodology (seven items: blinding in the biological assays performance, tested factor description, tissue sample conservation, description of the relevant test procedure of the biological factor, description of the negative and positive control procedures, test reproducibility control, definition of the level of positivity of the test), generalisability (six items: patient selection criteria, patients’ characteristics, initial investigation, treatment description, source of samples, number of unassessable samples with exclusion causes) and results analysis (four items: follow-up description, survival analysis according to the biological marker, univariate analysis of the prognostic factors for survival, multivariate analysis of the prognostic factors for survival) [67]. Each item was scored as follows: 2 points if the item was clearly defined in the article, 1 point if its description was incomplete or unclear and 0 point if it was not defined or if the definition was inadequate. The maximum theoretical score was 44 points. The final quality score was presented as a percentage, which was calculated using the following formula: sum of the total points divided by 44 and multiplied by 100. An optimal threshold has yet to be defined, but the cut-off of 75% of the quality scores represented half of the investigated studies. A higher percentage reflected a paper with better reporting quality.

### Exclusion criteria

The following studies were excluded from this meta-analysis: (1) studies published in a language other than English and incomplete studies; (2) case reports or animal studies; (3) studies involving only cellular models and lacking a clinical portion; (4) letters, case reports, commentaries, conference abstracts or review articles; (5) studies focusing on lncRNA genetic alterations, such as abnormal methylation patterns or polymorphisms; (6) studies whose HRs were based on data pertaining to multiple lncRNAs; (7) studies utilising only microarray analyses; and (8) studies lacking sufficient data for the calculation of HRs and corresponding 95% CIs. If data subsets were published in more than one article, only the most recent article was included in the analysis. Data were extracted independently by two authors (Yun Liu and Jifeng Feng) who reached a consensus regarding all data items.

### Statistical analysis

Statistical analysis was performed with Stata statistical software, version 12.0 (Stata Corp LP, College Station, TX, USA), and *P*-values less than 0.05 were considered statistically significant. Statistical heterogeneity between studies was assessed using the I^2^ statistic, and I^2^ > 50% signified the presence of significant heterogeneity [68]. A random- or fixed-effects model was used depending on the results of the heterogeneity analysis; if significant between-study heterogeneity was present, the random-effects model was used. However, if significant between-study heterogeneity was not present, the fixed-effects model was used. Pooled HRs and odds ratios (ORs) were extracted from the published data. In cases in which HRs could be directly obtained from a publication, we used crude HR values. In cases in which HRs and the corresponding 95% CIs were not directly reported in an included study, the survival data extracted from the corresponding Kaplan-Meier curves were used to estimate HRs. Stata 12.0 was used to determine the sensitivity of the studies. Publication bias was evaluated using Begg’s test [44], and a *P* < 0.05 was considered statistically significant.
